# Isostrain among tourism employees in Tunisia during COVID-19 pandemic: Sihem Ben Fredj

**DOI:** 10.1093/eurpub/ckac131.085

**Published:** 2022-10-25

**Authors:** A Maatouk, S Ben Fredj, N Zammit, R Ghammem, J Maatoug, S Boujebha, N Guesmi, B Zendah, H Ghannem, K Hadj Mabrouk

**Affiliations:** 1 Epidemiology Department, University Hospital Farhat Hached of Sousse, Sousse, Tunisia; 2 University of Sousse, Faculty of Medicine of Sousse, Sousse, Tunisia; 3 Groupement de Médecine du Travail de Sousse, Sousse, Tunisia

## Abstract

**Background:**

Psychological and social factors related to work activity can improve or deteriorate the physical and mental health of employees. Since the COVID-19 pandemic, the psychological well-being of workers has been strongly affected. Particularly, jobs with isostrain characterized by high work demands and low work control, coupled with low social support, place employees at highest risk for poor mental health. We aimed to assess the factors associated with isostrain among employees in the tourism sector in Sousse.

**Methods:**

A cross-sectional study was conducted between September and November 2020 among tourism workers belonging to 12 hotels and restaurants in Sousse, Tunisia, using a self-administered questionnaire. ‘Isostrain’ was assessed using the Karasek questionnaire. ‘Isostrain’ is a situation where there is a combination of ‘jobstrain’ (‘tension at work') and low social support (score below the median of the group). SPSS 20 software was used to analyze the data.

**Results:**

A total of 226 workers were included. The mean age was 38.2 ± 9.6 years. The sex ratio was 2.7. The majority of employees (64.8%) were working at workplace during COVID-19 lockdown. The prevalence of isostrain was 5.4%. Isostrain was reported by 5.7% of women (p = 1). All workers older than 50 years did not have isostrain (p = 0.6). Moreover, isostrain was found among 6.6% of workers with less than 5 years of work experience (p = 0.4), 4.8% of married employees (p = 0.7), and 7.6% of employees with a university education (p = 0.53).

**Conclusions:**

Isostrain can affect employees in the tourism sector. Social support should be promoted among workers to ensure good mental health.

**Key messages:**

• Prevalence of isostrain in tourism sector of Sousse during COVID-19 pandemic is quite high.

• Preventive strategies of mental health should be promoted at workplace in Tunisia.

